# Reelin and aromatase cooperate in ovarian follicle development

**DOI:** 10.1038/s41598-018-26928-x

**Published:** 2018-06-07

**Authors:** Maurice Meseke, Felicitas Pröls, Camilla Schmahl, Katja Seebo, Claas Kruse, Nicola Brandt, Lars Fester, Lepu Zhou, Roland Bender, Gabriele M. Rune

**Affiliations:** 1 Institute of Neuroanatomy, University Medical Center Hamburg-Eppendorf, Martinistr. 52, 20246 Hamburg, Germany; 2 Present Address: Department of Anatomy and Molecular Brain Research, Ruhr-University of Bochum, Universitätstrasse 150, 44780 Bochum, Germany; 3 Present Address: Institute of Anatomy II, University Medical Department of Cologne, Joseph-Stelzmann Str. 9, 50931 Köln, Germany; 4 Present Address: Institute of Anatomy, University of Oldenburg, Carl-von-Ossietzky Str. 9-11, 26129 Oldenburg, Germany

## Abstract

Reelin plays an important role in cerebral cortex development and synaptogenesis. In the hippocampus, the neurosteroid estrogen affects reelin expression. In this study we tested a potential crosstalk between estradiol and reelin, thus the possibility of a reelin-induced activation of the estradiol synthesizing enzyme aromatase. As a model system, we used ovaries, which express reelin and are a major source of estradiol. We found that in wild-type mice, reelin and aromatase are expressed in granulosa cells of growing follicles. The expression of reelin varies with the estrus cycle and is highest shortly before ovulation, when estradiol serum levels are at their maximum. In ovaries of reelin-deficient *reeler* mice, aromatase mRNA and protein are significantly reduced, as evidenced by real-time PCR, western blot analysis, and quantitative immunohistochemistry in granulosa cells of preovulatory follicles. In line with reduced estradiol synthesis, ovarian estrus cycle length is prolonged in *reeler* mice. Most importantly, treating cultured granulosa cells with recombinant reelin results in significant upregulation of aromatase mRNA and protein and increased secretion of estradiol into the supernatant. Our data provide evidence of a local increase of aromatase expression by reelin. Regarding reproduction, this crosstalk may contribute to follicular stability and counteract luteinization in ovaries.

## Introduction

Reelin is an extracellular matrix protein (ECM), and it has frequently been demonstrated that it is essential for cortical development. The absence of reelin results in migratory deficits of neurons^1,2^ and in impaired synaptogenesis in the brain^3–5^.

In the hippocampus, we have previously shown that reelin expression is estrogen-responsive^5^. Strong expression of estrogen receptor α has been found in Cajal-Retzius (CR) cells, which synthesize and secrete reelin. In hippocampal slice cultures, application of estradiol causes an increase in reelin expression in CR cells, which is abolished after blockade of estrogen receptors. Vice versa, inhibition of aromatase activity in hippocampal slice cultures by letrozole results in reduced reelin expression, suggesting that local estradiol synthesis in the hippocampus affects reelin expression.

It has been shown that reelin provides an inhibitory signal in the migration of gonadotropin-releasing hormone (GnRH) neurons^6^. GnRH neurons originate from the olfactory bulb and migrate to the basal forebrain during late embryonic development. Cariboni and colleagues^6^ demonstrated that the hypothalamus of developing and adult *reeler* mice contains a significantly reduced number of GnRH neurons due to altered migration. In the context of reproduction, GnRH induces the release of follicle stimulating hormone (FSH)/luteinizing hormone (LH) from the pituitary, which in turn promotes the release of peripheral gonadal hormones, such as estrogen and testosterone^7,8^. Serum concentrations of steroid hormones, in turn, eventually regulate the release of GnRH, but also FSH/LH, in a feed-back manner^9,10^; for review see^11^. GnRH and GnRH receptors (R), however, also influence ovarian development directly^12^. Accordingly, GnRH-Rs are strongly expressed in granulosa cells of advanced follicular stages^13,14^, and it was shown that GnRH is capable of stimulating estradiol synthesis in ovarian granulosa cells^15,16^. Hence, migratory deficits of GnRH neurons in *reeler* mice may result in altered levels of gonadal steroid hormone secretion in ovaries of *reeler* mice. Altered sex steroid levels, in turn, eventually may contribute to the impaired fertility in *reeler* mice, which was shown by Caviness *et al*., and Goffinet^17,18^.

Another possibility, however, could be that reelin, and aromatase, the final enzyme in estrogen synthesis, regulate each other. It has been shown that reelin promotes follicle development, which highly depends on ovarian estradiol synthesis^19^. To address this question of a direct crosstalk between reelin and aromatase we used ovaries and granulosa cell cultures, which have been shown to express reelin^20,21^ and which are a major source of estradiol. Hence, the female gonads, and in particular granulosa cells, appear to be particularly suitable for investigating a potential crosstalk between aromatase and reelin.

The results of these experiments demonstrate that aromatase activity depends on reelin expression. It is more likely that this interaction is responsible for the disturbances in the estrus cyclicity in *reeler* mice than irregularities in the hypothalamo-hypophyseal–gonadal axis of the mutant. Together with our previous findings in the hippocampus^5^, the results point to an interdependence between reelin expression and aromatase activity.

## Results

### Reelin is expressed in ovaries of mice

**Figure 1:**
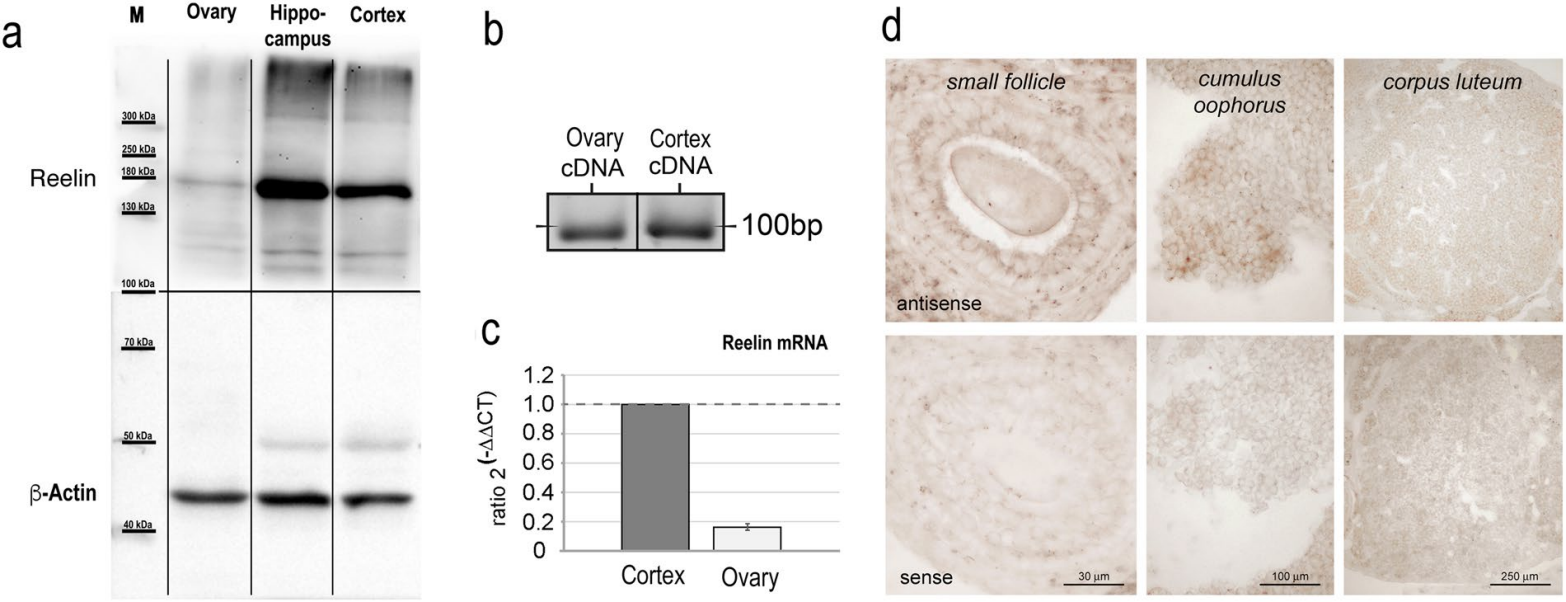
Reelin expression in ovaries of wild-type rat and mice. (**a**) Western blot bands of reelin protein in mouse ovaries, hippocampus, and cortex tissue. Independent of the investigated tissue, all tissues show a typical reelin band at 180 kDa size. (**b**) Gel bands of PCR amplification products of reelin mRNA with commercially available reelin primers in ovarian tissue compared to cortical tissue of the same mouse. (**c**) Real time quantification of reelin mRNA in cortical and ovarian tissue of 12-week-old mice with commercially available reelin primers demonstrated that both tissues contain reelin mRNA. However, expression is less prominent in the ovaries as compared to cortical tissue. (**d**) Non-radioactive *in situ* hybridization revealed distinct somatic signal in granulosa cells of small follicles (left panel) and of the cumulus oophorus (middle panel) in ovaries of 12-week-old mice, if antisense-probes against reelin mRNA were applied (top). No signal was detected in corresponding sections treated with the sense-probes (bottom). In corpora lutea (right panel), no difference between antisense- and sense-probe-treated sections was detectable.

Reelin could possibly act as a circulating protein from the blood stream that acts on the gonads, or it is expressed directly in the *gonads*. A prerequisite for reelin-induced aromatase expression is the presence of reelin protein in the ovaries. Western blot analyses of mouse ovarian tissue show that reelin protein is present in considerable amounts in ovaries and that the reelin protein shows the typical protein band of 180 kDa size, as numerously described in the literature for cortical tissue^3,5,22^ (Fig. 1a). Subsequent RT-PCR analyses substantiate that reelin synthesis, in fact, occurs in mouse ovarian tissue (Fig. 1b). Using real time PCR, reelin expression in the ovaries and in the cerebral cortex of the same mouse were quantified; this showed that the amount of reelin mRNA is more than 5 times lower in the ovaries than in cortex, yet substantial expression of reelin mRNA in mice ovaries is clearly evident (Fig. 1c). *In situ* hybridization against reelin was carried out using ovarian tissue sections to identify the cell types that express reelin in the ovaries of 12-week-old mice. The highest signal intensity of reelin mRNA was found in granulosa cells of growing follicles (Fig. 1d).

### Aromatase is downregulated in ovaries of *reeler* mice

Cyclic ovarian functions, such as ovulation, substantially depend on estradiol synthesis, and ovaries are the major source of estradiol in females. Accordingly, the enzyme aromatase cytochrome P450, which catalyzes the last step of estrogen biosynthesis and represents the rate-limiting irreversible aromatization of androgens to estrogens, is strongly expressed in ovaries, in particular in granulosa cells^23^.

**Figure 2:**
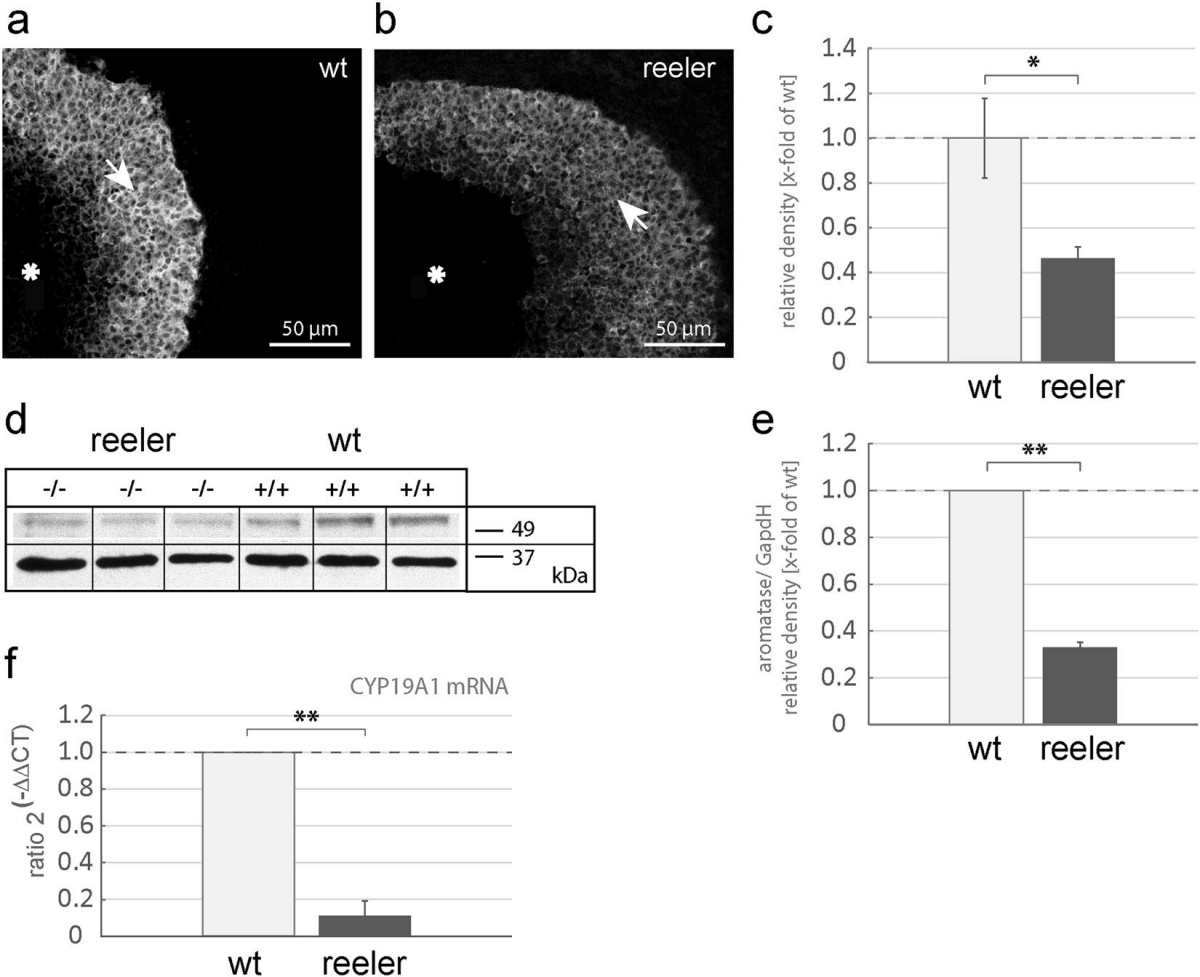
Aromatase expression in *reeler* mice. (**a**) Strong aromatase immunoreactivity was observable in granulosa cells (arrow) surrounding the antrum (star) of a tertiary follicle at proestrus (white arrow). (**b**) Aromatase immunoreactivity in the granulosa cell sheet (arrow) surrounding the antrum (star) of a corresponding *reeler* sibling was clearly less intensive. (**c**) Evaluation of aromatase signal intensities of wild-type and *reeler* mice in the granulosa cell sheet of tertiary follicles. Means are presented as x-fold values relative to the wild-type (relative density). The signal intensity of aromatase immunoreactivity is significantly reduced in the *reeler* (n = 3) compared to the wild-type (n = 3) mice. U-test, p = 0.04. (**d**) Western blotting bands of wild-type (+/+) compared to *reeler* (−/−) mice aromatase protein (55 kDa) in relation to a standard protein (GAPDH, 38 kDa). Full-length blot is accessible in supplementary information file. (**e**) Quantification of aromatase protein from ovaries of *reeler* mice (n = 3) normalized to wild-type (n = 3) protein endorsed a significant reduction in the U-test, p < 0.01. (**f**) Quantification of aromatase mRNA (CYP19A1) by means of real-time PCR via the ΔΔCT-method. Compared to wild-type mice (n = 3), aromatase mRNA was significantly reduced in *reeler* mice (n = 3), but was clearly detectable.

In agreement with the data in the literature, our immunocytochemical stainings of ovarian tissue sections showed a strong signal in the stratum granulosum of advanced antral follicles (Fig. 2a, white arrow), whereas in corresponding ovarian follicles of *reeler* mice, only very faint staining intensities were detectable (Fig. 2b, white arrow). Quantification of immunohistochemical stainings by confocal microscopy and subsequent image analysis of advanced antral follicles revealed a significant reduction of aromatase signal intensity (0.46 ± 0.05 × -fold of control; U-test; *p = 0.04) in *reeler* when compared to wild-type mice (Fig. 2c). We then prepared ovarian whole cell lysates of wild-type and *reeler* mice for western blot analyses to quantify aromatase protein levels (Fig. 2d,e). Figure 2d demonstrates the signal intensities of aromatase protein bands at a predicted size of 55 kDa of three different wild-type mice as compared to those bands of three different *reeler* mice of the same western blot experiment. Evaluation of the signal intensities of the aromatase bands confirms the reduction of aromatase protein (0.33 ± 0.02 × -fold of control) in the *reeler* mutant (Fig. 2e; U-test; **p < 0.01). Finally, a significant reduction by 90% (0.10 ± 0.02) in ovarian aromatase mRNA was demonstrated in the *reeler* compared to its wild-type siblings, as shown by taqman real-time qPCR (Fig. 2f; U-test; **p = 0.002).

### Coordinated expression of reelin and aromatase during the estrus cycle

**Figure 3:**
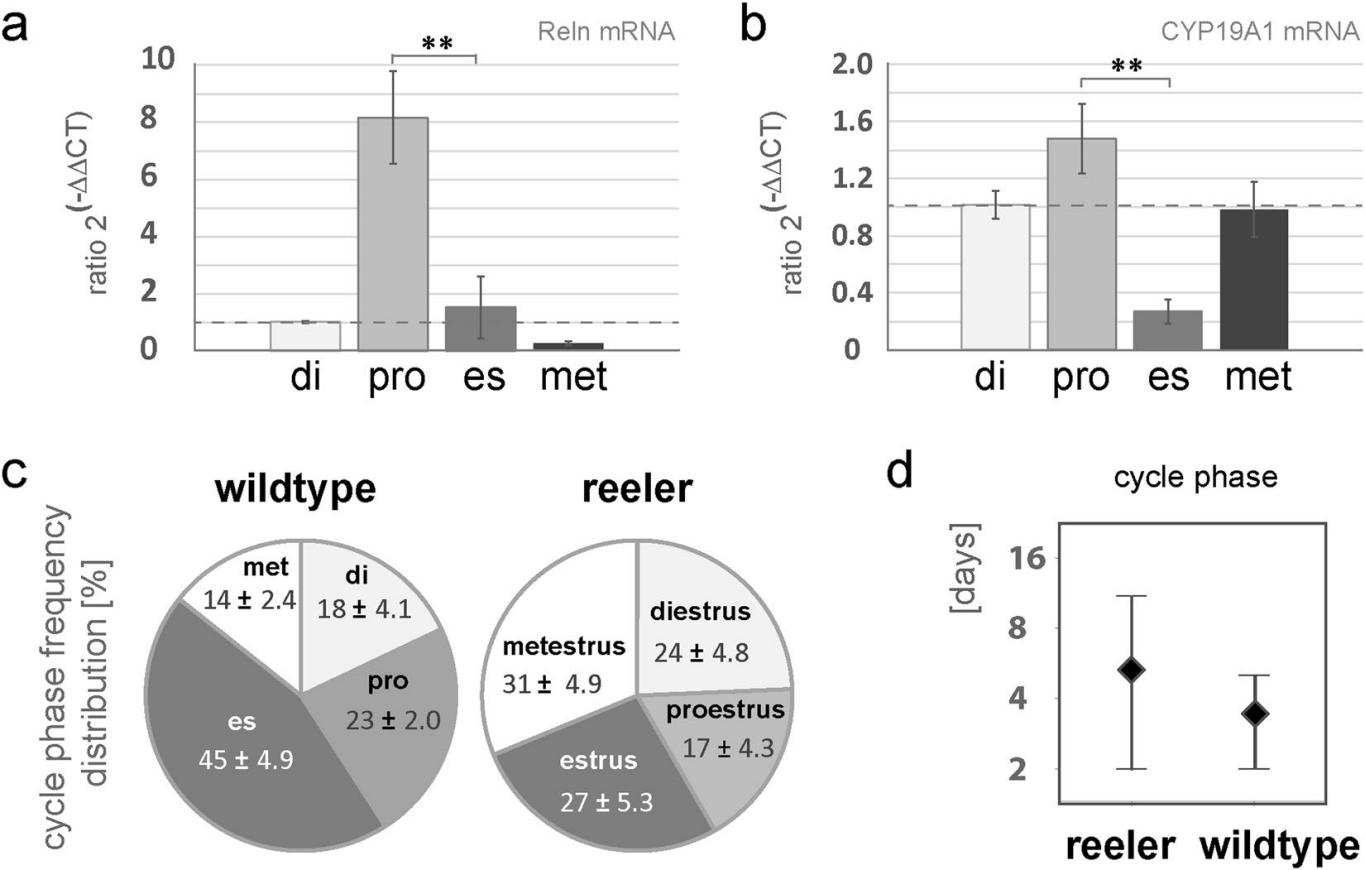
Estrus cycle of *reeler* mice. (**a**) Expression levels of reelin mRNA during the stages of the estrus cycle normalized to the diestrus. Highest expression levels of reelin mRNA were found in the proestrus phase of wild-type mice. The level of expression in the proestrus was significantly reduced in the estrus phase. Mann-Whitney-U-test, p = 0.01; n = 6 (diestrus), 14 (proestrus), 5 (estrus), 6 (metestrus). (**b**) Expression levels of aromatase (CYP19A1) mRNA during the stages of the estrus cycle normalized to the diestrus. Increased expression levels of aromatase mRNA were also found in the proestrus phase of wild-type mice, which are also significantly reduced in the estrus phase. U-test, p < 0.01; n = see above. (**c**) The pie chart depicts the percentage rate of the frequency distribution of the cyclic phases of *reeler* (n = 5) versus wild-type mice (n = 4). In the diestrus and proestrus phase, no differences were detectable, whereas in the metestrus phase an increase (~17%) and in the estrus phase a decrease (~18%) in the percentage of frequency of the cyclic phases is observable in the *reeler*. (**d**) The average cycle length in wild-type (n = 4) is 3.5 days, which is prolonged to 5.3 days in *reeler* (n = 5).

We further studied cyclic expression of reelin and aromatase mRNA in ovaries of 12-week-old mice at defined stages of the estrus cycle in order to address a potential crosstalk between aromatase and reelin. Figure 3a shows the expression of reelin mRNA during the estrus cycle, from diestrus (di) to proestrus (pro), followed by the estrus (es) and finally the metestrus (met) stage. Interestingly, we found an increase in reelin mRNA during the proestrus phase of approximately 8-fold compared to the diestrus phase. This increase in reelin mRNA correlates with an increase in aromatase mRNA during the proestrus stage, as well as a strong decrease in reelin mRNA and aromatase mRNA in the estrus phase compared to the proestrus stage (Fig. 3a; U-test; p = 0.01, 3b; U-test; p < 0.001). Data from the literature confirms that aromatase mRNA peaks during the proestrus phase^24,25^. Our finding would support the notion that estradiol increases reelin expression, confirming our previous results in hippocampal tissue^5^.

### Estrus cyclicity is disturbed in *reeler* mice

Since ovarian cyclicity is widely reflected by cyclic estradiol synthesis, and aromatase is downregulated in the ovaries of *reeler* mice, we studied the length of the estrus cycle in *reeler* mice by inspecting vaginal swabs. In 6 mutants and 5 wild-type mice the cyclic phase was determined daily over an averaged time period of 13 ± 9 days in the wild-type and 20 ± 10 days in the *reeler*. Based upon these data, the lengths of the various cyclic phases in *reeler* were compared to those of their wild-type siblings (Fig. 3c). Figure 3c shows the frequency distribution of the cycle phases within different stages of the estrus cycle. The frequency distribution of the cycle phase is highest for wild-type animals during the estrus phase (45% ± 4.9; Fig. 3c), but shifts in *reeler* to be highest during metestrus (31% ± 4.9; Fig. 3c), whereas other cycle stages such as proestrus and diestrus are not altered (Fig. 3c). In particular, the metestrus phase, when estradiol synthesis starts to increase to finally reach the preovulatory peak, is prolonged in *reeler* mice, and the later estrus phase is accordingly shorter than in wild-type. Hence, *reeler* mice show a disturbed gonadal cycle time, with a metestrus being extended from 14% (wild-type) up to 31% (*reeler* mutant, Fig. 3c) and the estrus is shortened from 45% (wt) to 27% in the *reeler* (Fig. 3c). The average cycle length in the wild-type is 3.5 days, with a maximum length of five and a minimum of two days (Fig. 3d), which is consistent with the data in the literature^26,27^. In contrast, *reeler* mice show an average cycle length of 5.3 days, with a maximum of 11 and a minimum of two days (Fig. 3d). Hence, the *reeler* has irregular cyclic phases.

### Reelin, but not GnRH, upregulates aromatase in granulosa cells

Our data on downregulated aromatase expression and disturbed estrus cyclicity in *reeler* mice suggest that reelin regulates estradiol synthesis. GnRH has to be considered as a further player. Due to the migratory deficit of GnRH neurons in *reeler* mice^6^, estradiol synthesis might be disturbed by lack of GnRH in *reeler* mice and accordingly, aromatase expression would be downregulated.

**Figure 4:**
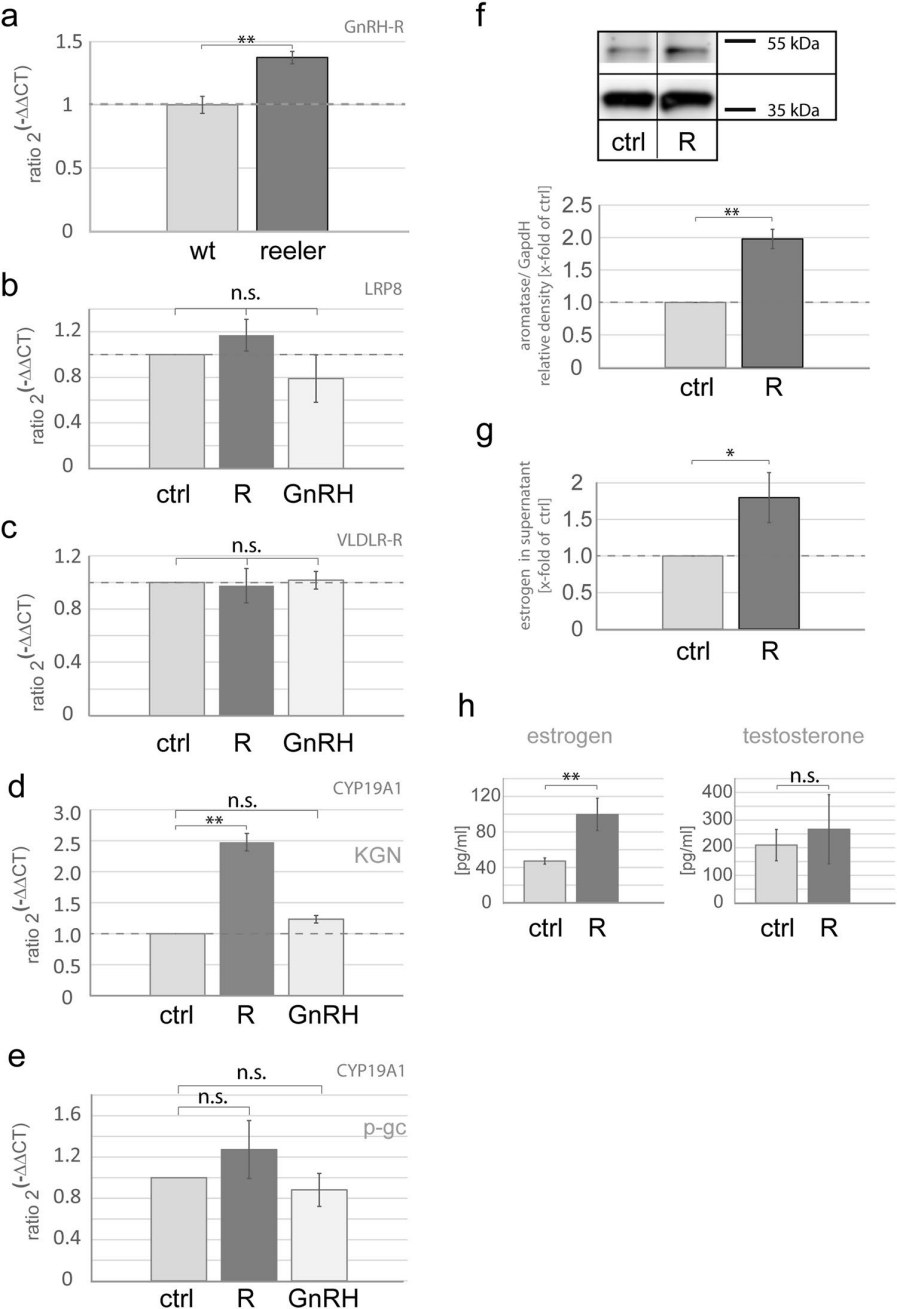
Reelin, but not GnRH, stimulates aromatase expression and activity. (**a**) Real-time PCR demonstrates a significant increase in the GnRH-Receptor (GnRH-R) expression level of about 28% in the ovaries of *reeler* mice (reeler: 1.28; n = 5) as compared to wild-type mice (n = 5). Mann-Whitney-U-test, p = 0.006. (**b**) Neither reelin (R) (n = 5) nor GnRH (n = 5) stimulation resulted in a significant change of LRP8 mRNA of KGN cells after 2 h of stimulation compared to control (n = 3). (**c**) VLDLR mRNA is not altered after 2 h of stimulation with reelin (R) (n = 5) or GnRH (n = 5) in KGN-cells. (**d**) Aromatase mRNA (CYP19A1) is increased 2.5 times as much compared to the control (n = 3) in KGN cells after reelin (R = 2.48; n = 5) treatment for 2 h. No effect was observed by stimulation with GnRH (n = 8). Kruskal-Wallis-ANOVA, post-hoc Bonferroni, p < 0.01. (**e**) Aromatase mRNA (CYP19A1) is increased, but not significantly changed compared to control (n = 9) in primary granulosa cells after reelin (R = 1.27; n = 9) treatment for 2 h. No effect was observed by stimulation with GnRH (n = 8). Kruskal-Wallis-ANOVA, p = 0,11. (**f**) Analysis of aromatase protein by western blotting after 24 h of stimulation with reelin. The upper picture shows aromatase bands of an exemplary western blot with a predicted size of about 55 kDa under non-stimulated and stimulated conditions. Reelin (R = 1.98; n = 11) stimulation significantly increased aromatase protein about twice as much as compared to the control (n = 4). Full-length blot is accessible in supplementary information file. (**g**) RIA measurement of estrogen content in the supernatant of KGN cells after 24 h of stimulation. Reelin (R = 1.80; n = 19) stimulation significantly increased the amount of estrogen in the supernatant as compared to the control (n = 9). U-test, p = 0.048. (**h**) Reelin treatment for 24 h significantly increased estrogen content in the supernatant from 47 pg/ml to 99,6 pg/ml. T-test; *p = 0.036; (n = 7). Testosterone levels were not significantly altered by reelin treatment within 24 h, T-test; p = 0.71 (n = 7).

In agreement with previously published findings^14,28,29^, we found expression of GnRH-R mRNA in the ovaries of both WT and *reeler* mice, supporting the idea that ovaries are in fact responsive to GnRH (Fig. 4a). Remarkably, GnRH-R expression was even significantly higher in *reeler* mice (Fig. 4a; U-test; **p < 0.001). In order to investigate whether GnRH induces aromatase expression, we stimulated granulosa cells with GnRH or with recombinant reelin protein. The presence of the reelin receptors ApoER2 (LRP8) and VLDRR has been shown in primary granulosa cell cultures^19^, and in this study we show that this also holds true for KGN cells (Fig. 4b,c). No significant effect on ApoER2 and VLDRR mRNA expression was seen after treatment with GnRH or reelin (Fig. 4b,c). However, after application of reelin to the medium of KGN cells for 2 hours, an increase in aromatase mRNA (CYP19A1) was detected in KGN cells and in primary granulosa cells, as revealed by real-time PCR, whereas no effect was seen after treatment with GnRH (Fig. 4d,e). The increase in aromatase mRNA was significant in KGN cells (Fig. 4d; Kruskal-Wallis ANOVA **p < 0.001, post-hoc Bonferroni **p < 0.001) and was seen in tendency in primary granulosa cells (Fig. 4e). At the protein level, we were also able to demonstrate that 2 h incubation of KGN cells with recombinant reelin protein resulted in a significant increase in aromatase protein (Fig. 4f; T-test; **p = 0.009).

Measurements of estradiol content in the medium using either RIA (KGN cells; Fig. 4g) or an ELISA-kit (primary granulosa cells; Fig. 4h) underscore that reelin affects estradiol synthesis in KGN cells and in primary granulosa cells. The amount of estradiol in the supernatant of KGN cells was significantly elevated after reelin treatment for 24 h (Fig. 4g; U-test; *p = 0.048). In granulosa cells, reelin treatment doubled the amount of estrogen in the supernatant from 47 pg/ml to approximately 100 pg/ml (Fig. 4h, T-test; *p = 0.036). Testosterone levels were not significantly affected in response to application of reelin for 24 h (Fig. 4h, T-test; p = 0.71).

## Discussion

In this study, we demonstrate that the expression of reelin and aromatase is coordinated in the ovaries, and most importantly, that reelin activates estradiol synthesis in ovarian granulosa cells. Reduced estradiol levels, and as a potential consequence, irregularities in the estrus cyclicity in *reeler*, may contribute to reduced fertility of these mutants.

### Impaired fertility in reeler mice

It has long been known that the fertility of *reeler* mice is reduced^30^. Difficulties in breeding *reeler*, together with the finding that in *reeler* the number of GnRH neurons in the hypothalamus is reduced^6^, suggests that irregularities in the hypothalamo-hypophyseal-gonadal axis account for the disturbed reproductive functions in *reeler* mice. At a first glance, morphological alterations in the testes support this view. Cariboni *et al*.^6^ found a reduction in the number of seminiferous tubules in male gonads. Hyperplasia of Leydig cells, a typical feature after insufficient GnRH secretion^31^, however, was not seen. Thus, the reduction in seminiferous tubule number is not likely to result from GnRH deficiency, but may result from local disturbances of spermatogenesis in *reeler* mice. Regarding the existence of GnRH-Rs, which we found in ovaries of both *reeler* and wild-type mice, and which have previously been reported as being present in granulosa cells^14,28^, a direct response of ovarian tissue and granulosa cells to GnRH might be assumed. In contrast to previous reports^15,16^, however, this was not the case, but reelin that was found to have an effect on aromatase expression in granulosa cells.

Consistently, estrus cyclicity, which we tested by monitoring the estrus cycles of *reeler* mice, is disturbed in *reeler*. Estrus cyclicity widely depends on cyclic synthesis of progesterone and estradiol, the latter being preferentially synthesized in granulosa cells. Our data show that the average cycle length is prolonged, and as a consequence, the frequency of ovulations must be reduced. Increased synthesis of LH, as shown by Lombadero *et al*.^32^, which may represent a feedback response to low estradiol levels, is obviously not able to compensate for reduced estradiol synthesis due to lack of reelin in the ovaries of *reeler* mice. Hence, reelin deficiency in the *reeler* mutant and lack of reelin-induced aromatase expression very likely account for irregular estrus cyclicity and may contribute to impaired fertility.

### Reelin signaling to induce estrogen synthesis

This is the first study to show that reelin influences estradiol synthesis. However, reelin is an ECM protein, and numerous earlier studies have shown that ECM proteins are able to influence steroidogenesis in the gonads and in gonadal cells *in vitro*^33–36^; for review see^37,38^.

It was recently shown that reelin signaling promotes granulosa cell proliferation in chicken ovarian follicles^19^ via activation of ApoE2 and VLDR receptors and subsequent dab1 phosphorylation. Ovarian follicle development, however, is also highly dependent on estradiol synthesized in granulosa cells^23^. Since reelin stimulates estradiol synthesis, a synergistic action of reelin and estradiol in follicle development is probable. Follicle development is largely a function of granulosa cell proliferation and differentiation. Since aromatase is downregulated in single granulosa cells of growing follicles in *reeler*, as compared to WT animals, and reelin increases aromatase mRNA and protein, the proliferative effect of reelin appears to be mediated by enhanced aromatase expression and estradiol synthesis.

Regarding the expression pattern of reelin, it is seen in granulosa cells but not in corpora lutea; this confirms previous studies by Fayad *et al*.^39^ and Eresheim and coworkers^19^. It appears that reelin plays a role in follicle development by stabilizing the follicle. Reelin binds to ApoE2R and VLDR receptors, which results in the phosphorylation of dab1, an adapter protein interacting with the cleaved Notch intracellular domain^19,40–42^. Notch signaling is required for follicle development and fertility and is, similar to reelin, expressed in the cells of growing follicles. Several lines of evidence indicate that Notch signaling in the ovary is necessary for granulosa cell proliferation, as shown for reelin^19^ and normal follicle development^43–47^. In the context of steroidogenesis, however, it was shown that Notch signaling stimulates progesterone production in luteal cells^48^, while steroidogenesis in granulosa cells of small follicles is suppressed, and it was proposed that Notch signaling inhibits FSH-induced steroidogenic gene expression^49^. In contrast, in this study we found that aromatase mRNA is upregulated in granulosa cells, which results in enhanced estradiol secretion, suggesting that reelin-induced Notch signaling is unlikely to participate in the control of estradiol synthesis in granulosa cells.

Alternatively, dab1 phosphorylation induced by reelin binding to ApoER2 and VLDLR activates PI3K and LIM kinase1, which in turn results in the phosphorylation of cofilin^50^. Phosphorylation renders cofilin unable to depolymerize F-actin, and active cofilin/ADF is involved in ovary development and oogenesis^51–53^. Karlsson *et al*.^54^ showed that active cofilin is required to initiate luteinization, thus the transformation of the follicular wall into a corpus luteum, which is paralleled by a rise in progesterone secretion and substantial rearrangement of cytoskeleton^47^. This rearrangement of the cytoskeleton includes activation (dephosphorylation) of cofilin. Importantly, inactive cofilin caused a 70% decrease in LH-stimulated progesterone secretion into the supernatant. Based upon these results, it seems likely that reelin-induced phosphorylation of cofilin in growing follicles prevents luteinization until the LH surge is triggered by increasing estradiol synthesis. The finding that ApoER2/LRP8, which binds reelin, is maximally expressed in granulosa cells of dominant follicles and is downregulated upon hCG/LH stimulation, supports this hypothesis^21^. In addition, the downregulation of ApoER2 is paralleled by decreased aromatase expression^55^. Finally, ApoE deficient mice, mutants that lack the natural ligand of the ApoER2, show decreased aromatase expression^56^. Hence, reelin by phosphorylating cofilin, likely counteracts luteinization by stabilizing granulosa cells.

As shown in this study, estradiol and reelin may act synergistically by converging at the same intracellular cascades, thus producing similar effects. Thus, expression of both reelin and aromatase is highest during proestrus of WT mice. Estradiol and reelin share common signaling molecules such as Notch^5,57^, cofilin^58,59^ and PI3K/GSK3^60,61^. In this study we provide evidence that reelin increases aromatase expression and estradiol synthesis, hence pointing to a positive feed-back mechanism, which needs to be analyzed in detail in future comparative studies on the ovaries and hippocampus.

## Material and Methods

### Animals

*Reeler* mice (B6C3Fe-a/a-relnrl; Jackson Laboratory) and sibling wild-type (wt) mice were reared in accordance with the animal care guidelines of the University of Hamburg. Genotypes were verified by PCR analysis of genomic DNA. Ovaries of 12-week-old *reeler* mice and sibling wild-type mice were used for real time PCR, western blot and for immunostaining of tissue sections. Twelve-week-old Wistar rats (Institute of Anatomy, Hamburg, Germany) were maintained under controlled conditions; water and food were available ad libitum and taken for *in situ* hybridization experiments. All experiments were carried out in accordance with the German law on the use of laboratory animals (German Animal Welfare Act; project number ORG 470 by the “Amt für Verbraucherschutz, Lebensmittelsicherheit und Veterinärwesen”). All methods described below were accomplished in accordance with the relevant guidelines and regulations. Estrus cyclicity is mirrored by changes of the cellular composition in vaginal smears^24,25^. The cyclic stage was determined by a vaginal smear that was stained using standard procedures^62,63^. When comparing *reeler* with wild-type animals, only animals in the proestrus stage were taken for experiments. Firstly, these animals show the highest expression of aromatase in the ovaries^24^ and secondly, these animals therefore possess a comparable hormonal status (Figs 2, 4). Sexual maturity and cyclic regularity were controlled, at the earliest, 8 weeks postnatal, again by vaginal smear.

### Cell culture

The human ovarian granulosa-like tumor cell line, designated KGN^64^, was taken for aromatase mRNA studies or western blot experiments. KGN cell cultures were cultured as described by Nishi and colleagues^64^ with DMEM/F-12 (5%) (HAM), Pen-Strep (1%) and incubated at 37 °C and 5% CO_2_ in a humidified incubator. The first experiment was performed after three times passage of KGN cells, and the cells were then constantly kept in culture for following experiments. For each experiment, cells were seeded initially with a ratio of 1:200.000 cells per dish in a 6-well plate and stimulation was done on DIV 3.

Primary granulosa cell cultures were prepared from 12-week-old female mice. The ovaries were removed, cut open and follicles picked out without discrimination of developmental age. The follicles were washed in PBS and then digested with 1 mg/ml collagenase type IV (Sigma) for 15 min at 37 °C in DMEM, 10% FCS and Pen-Strep (1%). Afterwards, the granulosa cell suspension was centrifuged, washed with PBS three times and resuspended in DMEM, 10% FCS, Pen-Strep (1%) and seeded in six-well dishes. On DIV 3, stimulation with reelin was performed for 24 h for estradiol determination in the supernatant, or stimulation on DIV 4 for 2 h with reelin for aromatase RNA quantification.

### Preparation of reelin-conditioned medium and control medium

HEK-293 cells were stably transfected with a plasmid containing full-length reelin cDNA^65^ or, as a control, with the same plasmid expressing GFP. Serum-free supernatants containing secreted reelin and control supernatants not containing reelin (control = mock) were collected as previously described^66^.

### Antibodies and reagents

The following primary antibodies were used for immunocytochemistry or western blotting: rabbit polyclonal anti-aromatase (1:1000)^67–69^, mouse monoclonal anti-aromatase (1:250; Acris SM2222P, USA), or mouse monoclonal anti-reelin (G10, MAB 5364, Merck Millipore, Germany). Secondary antibodies were Alexa Fluor 488-conjugated goat anti-rabbit (1:500) and/or Alexa Fluor 555-conjugated goat anti-mouse (1:500; Thermo Fisher, USA). Nuclei were counterstained with the fluorescent dye DAPI (1:10 000; Molecular Probes, USA).

Cell cultures stimulated with GnRH1 (Sigma-Aldrich, USA) were used in a final concentration of 10^−7^ M in the culture medium.

### Immunohistochemistry

After removal, the ovaries were placed in liquid nitrogen and cut frozen. 12 µm sections were cut on a cryomicrotome (HM560) and fixed afterwards with acetone and subsequently, after washing, with 4% paraformaldehyde. Fixation, as well as the following steps, were performed on a microscopic slide inside a humid chamber. Tissue sections were blocked with 10% NGS-PBS (Sigma-Aldrich, USA) for 30 min, washed, and then incubated overnight in the primary antibody dissolved in a 10% NGS-PBS solution. After washing with PBS, secondary antibody binding (dissolved only in PBS) was performed for one hour on a shaker at room temperature. Finally, after washing, the nuclei were stained with Dapi (4′,6-Diamidino-2-phenylindol) and cryosections were mounted in DAKO (Agilent Technologies, USA). Images were captured with a confocal Zeiss LSM (Axiovert 100, MikroSystems LSM) by using a Zeiss Plan-Neofluar (40x, numerical aperture 0.75) objective lens. Altogether, we analyzed 47 optical slices from (in the mean) 8 different preovulatory follicles of 3 reeler mice and (in the mean) 38 optical slices from (in the mean) 7 follicles of 3 different wild type mice. Quantification of signal intensity (densitometry) was achieved using ImageJ software (NIH, USA). For calculation of relative values, the mean of the wild-type values was set at 1 and the values determined in the *reeler* were related to it and presented as x-fold values of control.

### Western blot

Ovaries from 12-week-old female mice were removed, frozen on liquid nitrogen and consecutively homogenized in ice cold RIPA lysis buffer [1% NP40, 0.1% SDS, 0.5% Na-deoxcholate, protease inhibitor and phosSTOP (Roche, Switzerland)]. Cell cultures were lysed in ice cold RIPA and frozen on liquid nitrogen; 15–30 µg of each sample were diluted in water and 5 × Laemmli buffer [62.5 mm tris base (ph 6.8); 2% SDS; 10% glycerol; 5% 2-mercaptoethanol; 0,001% bromphenolblau] added to a final volume of 12.5 µl. They were heated to 95 °C for 5 min and then immediately cooled on ice. The samples were separated on 10% polyacrylamide gel by gel electrophoresis (Invitrogen, Germany) in Laemmli running buffer [10% SDS, 3% tris base; 14% glycin] and transferred electrophoretically to polyvinylidene fluoride (PVDF) membranes with transfer buffer [0,02% SDS; 0,015% tris base; 0,08% glycin]. For blotting, the membranes were blocked with 5% bovine serum albumin (BSA) in phosphate-buffered saline (PBS) at room temperature (RT) for 1 h and incubated with primary antibodies in blocking solution at 4 °C overnight. Secondary antibodies, which were conjugated with an alkaline phosphatase, were incubated for one hour at RT (Western Breeze Chemiluminescent Immunodetection Kit, Invitrogen, Germany). The immunoreaction was visualized by enhanced chemiluminescence (FUSION-SL4 advanced imaging system; Vilber Lourmat, Belgium) and signal intensity (densitometry) was quantified using ImageJ software (NIH, USA). The results are presented as x-fold relative differences to control.

### Primer

For a qualitative detection of reelin mRNA in mice ovaries, mice-specific TaqMan probes (Thermo Fisher, USA) for reelin (RELN; Mm00465200) were used.

### Real-time qPCR using TaqMan probes

Tissue was homogenized by breaking deep- frozen tissue into small pieces, followed by up and down pipetting with decreasing needle size (20 G–27 G needles, Braun) in RLT buffer containing RNAse inhibitor (Qiagen, Germany). Final tissue homogenisation of either mice ovaries or KGN cells was achieved using QIAshredder column (Qiagen) for PCR analysis, according to the instructor’s manual. The RNeasy Mini Kit (Qiagen) was used for mRNA isolation, including digestion with DNase I (Qiagen). Elution of mRNA was done in RNase free water. RT^2^ First Strand Kit (Qiagen) was used to synthesise cDNA, following the instructor’s manual. Mice-specific TaqMan probes (Thermo Fisher, USA) for aromatase (Cyp19a1; Mm00484049_m1), reelin (RELN; Mm00465200) and Hypoxanthin-Guanin-Phosphoribosyltransferase (Hprt; house-keeping gene; Mm03024075_m1) or human specific TaqMan probes for aromatase (Cyp19a1; Hs00903411_m1), Hprt (Hs01003267_m1), VLDLR (Hs01045922_m1) and LRP8 (Hs00182998_m1) were obtained from Thermo Fisher Scientific, and a StepOnePlus^TM^ Real-Time PCR System with 96-well plates was used. mRNA levels were calculated using the 2^(−ΔΔC(T))^ method according to Livak and Schmittgen^70^.

### *In situ* hybridization (ISH)

For ISH, 12-week-old female wild-type mice were deeply anesthetized with a ketamine-xylazine mixture (ketamine 12 mg/ml, xylazine 0.16% in saline, i. p.), then transcardially perfused with 4% PFA and the ovaries were removed. After cryoprotection (25% sucrose) for 4 hours, the ovaries were cryosectioned (16–22 µm), the sections transferred to RNase-free glass slides and subjected to non-radioactive *in situ* hybridization. Digoxigenin (DIG)-labeled antisense and sense cRNA-probes were transcribed *in vitro* from mouse reelin cDNA^71^ using T3- and T7-RNA polymerases, respectively (Boehringer, Germany). Probes were used to detect reelin mRNA (antisense) or as non-specific control (sense). Specificity of synthesized probes was tested by *in situ* hybridization against reelin, identifying Cajal-Retzius cells in mouse hippocampal tissue. For hybridization, slide-mounted sections were washed with 2 × SSC (0.3 M NaCl, 0.03 M Na-citrate) for 30 min, then incubated for 30 min with 2 × SSC/prehybridization solution (1:1) and transferred to a humid chamber for prehybridization (1 hour at 47 °C). The prehybridization solution consisted of 50% formamide, 4 × SSC buffer, 5 × Denhardt’s solution, 5% dextran sulfate, 100 μg/ml yeast tRNA, and 100 μg/ml salmon sperm DNA. For hybridization, DIG-labeled RNA probes (antisense or sense) were added, and sections were incubated at 47 °C for at least 12 hours. For all steps, RNase-free solutions were used. After hybridization, sections were washed in 2 × SSC (2 × 15 min at room temperature), 50% formamide/2 × SSC (20 min at 57°), 50% formamide/0.1 × SSC (20 min at 57°), and 0.1 × SSC (20 min at 57°). Subsequently, hybrid molecules were detected with an anti-DIG serum tagged with alkaline phosphatase and binding was visualized with 4-nitro blue tetrazolium chloride and 5-bromo-4-chloro-3-indolyl phosphate, according to the protocol of the manufacturer (Roche Diagnostics, Germany).

### Radioimmunoassay for estradiol

KGN cultures were preincubated for 15 min with 10^−7^ testosterone (Sigma) as a substrate for granulosa cell aromatase. Culture supernatants of the medium treated with reelin and mock-treated KGN cultures were collected after 24 h of treatment. 5 ml of the culture supernatant were collected for each condition. A commercially available radioimmunoassay kit (DIA Source: E2-RIA-CT KIP0629) was used for measurement of estrogen (E2). For RIA, 50 µl of culture supernatant were applied and E2 concentrations were calculated from the results of these measurements and presented as x-fold values of control. RIA was analyzed on a gamma counter (RIASTAR). To calculate the relative values, the mean of the steroid concentrations determined in the medium collected from control cultures was set at 1 and the values determined in the treatment groups were related to it and presented as x-fold values of control. The mean value for estrogen determined in the supernatant of the KGN cells was in the range of 581 pg/ml under control conditions (n = 9) and 699 pg/ml after reelin stimulation (n = 19).

### ELISA for estradiol and testosterone content determination

Primary granulosa cell cultures from 12-week-old mice were preincubated for 15 min with 10^−7^ testosterone (Sigma) as a substrate for granulosa cell aromatase. Culture supernatants of the medium treated with reelin and mock-treated granulosa cell cultures were collected after 24 h of treatment. 2 ml of the culture supernatant were collected for each condition. For determination of estrogen or testosterone in the supernatant, a commercially available ELISA kit (17β-estradiol Saliva ELISA RE52601, IBL, Hamburg, Germany; Tetosterone Saliva ELISA RE52631, IBL, Hamburg, Germany) was used. Photometric analysis was done with the ELISA-Reader Dynatech Laboratories MRX (Dynex-Technologies, Denkendorf, Germany).

### Statistics

The results show mean values ± SEM. Means were tested for normal distribution and variance homogeneity. Depending on the experimental group, the unpaired *t-*test was chosen using SPSS software. In the case of non-normal distribution of the raw data, the Mann- Whitney-U-Test or Kruskal-Wallis ANOVA, with a following Bonferroni post-hoc test, was used for statistics. For all statistical test procedures, the level of significance was set at p < 0.05.

## Electronic supplementary material

Supplementary Dataset 1

## References

[1] Curran T, D’Arcangelo G (1998). Role of reelin in the control of brain development. Brain Res Brain Res Rev.

[2] Forster E (2006). Recent progress in understanding the role of Reelin in radial neuronal migration, with specific emphasis on the dentate gyrus. Eur J Neurosci.

[3] D’Arcangelo G (1999). Reelin is a ligand for lipoprotein receptors. Neuron.

[4] Herz J, Chen Y (2006). Reelin, lipoprotein receptors and synaptic plasticity. Nat Rev Neurosci.

[5] Bender RA (2010). Roles of 17ss-estradiol involve regulation of reelin expression and synaptogenesis in the dentate gyrus. Cereb Cortex.

[6] Cariboni A (2005). Reelin provides an inhibitory signal in the migration of gonadotropin-releasing hormone neurons. Development.

[7] Conti M (2002). Specificity of the cyclic adenosine 3′,5′-monophosphate signal in granulosa cell function. Biol Reprod.

[8] Tonetta SA, diZerega GS (1989). Intragonadal regulation of follicular maturation. Endocr Rev.

[9] Steinkampf MP, Mendelson CR, Simpson ER (1987). Regulation by follicle-stimulating hormone of the synthesis of aromatase cytochrome P-450 in human granulosa cells. Mol Endocrinol.

[10] Zeleznik AJ (1981). Premature elevation of systemic estradiol reduces serum levels of follicle-stimulating hormone and lengthens the follicular phase of the menstrual cycle in rhesus monkeys. Endocrinology.

[11] Cariboni A, Maggi R, Parnavelas JG (2007). From nose to fertility: the long migratory journey of gonadotropin-releasing hormone neurons. Trends Neurosci.

[12] Maggi R (2005). Factors involved in the migration of neuroendocrine hypothalamic neurons. Arch Ital Biol.

[13] Bauer-Dantoin AC, Jameson JL (1995). Gonadotropin-releasing hormone receptor messenger ribonucleic acid expression in the ovary during the rat estrous cycle. Endocrinology.

[14] Choi JH, Gilks CB, Auersperg N, Leung PC (2006). Immunolocalization of gonadotropin-releasing hormone (GnRH)-I, GnRH-II, and type I GnRH receptor during follicular development in the human ovary. J Clin Endocrinol Metab.

[15] Parinaud J, Beaur A, Bourreau E, Vieitez G, Pontonnier G (1988). Effect of a luteinizing hormone-releasing hormone agonist (Buserelin) on steroidogenesis of cultured human preovulatory granulosa cells. Fertil Steril.

[16] Janssens RM (2000). Direct ovarian effects and safety aspects of GnRH agonists and antagonists. Hum Reprod Update.

[17] Caviness VS, Korde MG, Williams RS (1977). Cellular events induced in the molecular layer of the piriform cortex by ablation of the olfactory bulb in the mouse. Brain Res.

[18] Goffinet AM (1984). Phylogenetic determinants of radial organization in the cerebral cortex. Z Mikrosk Anat Forsch.

[19] Eresheim C, Leeb C, Buchegger P, Nimpf J (2014). Signaling by the extracellular matrix protein Reelin promotes granulosa cell proliferation in the chicken follicle. J Biol Chem.

[20] Ikeda Y, Terashima T (1997). Expression of reelin, the gene responsible for the reeler mutation, in embryonic development and adulthood in the mouse. Dev Dyn.

[21] Fayad T, Lefebvre R, Nimpf J, Silversides DW, Lussier JG (2007). Low-density lipoprotein receptor-related protein 8 (LRP8) is upregulated in granulosa cells of bovine dominant follicle: molecular characterization and spatio-temporal expression studies. Biol Reprod.

[22] Lambert de Rouvroit C (1999). Reelin, the extracellular matrix protein deficient in reeler mutant mice, is processed by a metalloproteinase. Exp Neurol.

[23] Boon WC, Chow JD, Simpson ER (2010). The multiple roles of estrogens and the enzyme aromatase. Prog Brain Res.

[24] Turner KJ (2002). Development and validation of a new monoclonal antibody to mammalian aromatase. J Endocrinol.

[25] Stocco C (2008). Aromatase expression in the ovary: hormonal and molecular regulation. Steroids.

[26] Pickles AC (1987). Current therapy in theriogenology. Equine Veterinary Journal.

[27] Allen E (1923). Racial and familial cyclic inheritance and other evidence from the mouse concerning the cause of oestrous phenomena. American Journal of Anatomy.

[28] Cheng JC, Klausen C, Leung PC (2013). Overexpression of wild-type but not C134W mutant FOXL2 enhances GnRH-induced cell apoptosis by increasing GnRH receptor expression in human granulosa cell tumors. PLoS One.

[29] Maggi, R. *et al*. GnRH and GnRH receptors in the pathophysiology of the human female reproductive system. *Hum Reprod Update* **22**, 10.1093/humupd/dmv059 (2016). 10.1093/humupd/dmv059 26715597

[30] Caviness VS, So DK, Sidman RL (1972). The hybrid reeler mouse. J Hered.

[31] Forni PE, Wray S (2015). GnRH, anosmia and hypogonadotropic hypogonadism–where are we?. Front Neuroendocrinol.

[32] Lombardero M, Kovacs K, Horvath E, Salazar I (2007). Hormonal and morphological study of the pituitaries in reeler mice. Int J Exp Pathol.

[33] Huet C, Monget P, Pisselet C, Monniaux D (1997). Changes in extracellular matrix components and steroidogenic enzymes during growth and atresia of antral ovarian follicles in the sheep. Biol Reprod.

[34] Le Bellego F, Pisselet C, Huet C, Monget P, Monniaux D (2002). Laminin-alpha6beta1 integrin interaction enhances survival and proliferation and modulates steroidogenesis of ovine granulosa cells. J Endocrinol.

[35] Clavero A (2004). Expression of integrin fraction and adhesion molecules on human granulosa cells and its relation with oocyte maturity and follicular steroidogenesis. J Assist Reprod Genet.

[36] Harlow CR, Bradshaw AC, Rae MT, Shearer KD, Hillier SG (2007). Oestrogen formation and connective tissue growth factor expression in rat granulosa cells. J Endocrinol.

[37] Berkholtz CB, Shea LD, Woodruff TK (2006). Extracellular matrix functions in follicle maturation. Semin Reprod Med.

[38] Nagyova E (2012). Regulation of cumulus expansion and hyaluronan synthesis in porcine oocyte-cumulus complexes during *in vitro* maturation. Endocr Regul.

[39] Lussier AL, Weeber EJ, Rebeck GW (2016). Reelin Proteolysis Affects Signaling Related to Normal Synapse Function and Neurodegeneration. Front Cell Neurosci.

[40] Weeber EJ (2002). Reelin and ApoE receptors cooperate to enhance hippocampal synaptic plasticity and learning. J Biol Chem.

[41] Forster E (2010). Emerging topics in Reelin function. Eur J Neurosci.

[42] D’Arcangelo G (2005). The reeler mouse: anatomy of a mutant. Int Rev Neurobiol.

[43] Hahn KL, Johnson J, Beres BJ, Howard S, Wilson-Rawls J (2005). Lunatic fringe null female mice are infertile due to defects in meiotic maturation. Development.

[44] Trombly DJ, Woodruff TK, Mayo KE (2009). Suppression of Notch signaling in the neonatal mouse ovary decreases primordial follicle formation. Endocrinology.

[45] Zhang QG (2011). C terminus of Hsc70-interacting protein (CHIP)-mediated degradation of hippocampal estrogen receptor-alpha and the critical period hypothesis of estrogen neuroprotection. Proc Natl Acad Sci USA.

[46] Xu J, Gridley T (2012). Notch Signaling during Oogenesis in Drosophila melanogaster. Genet Res Int.

[47] Vanorny DA, Prasasya RD, Chalpe AJ, Kilen SM, Mayo KE (2014). Notch signaling regulates ovarian follicle formation and coordinates follicular growth. Mol Endocrinol.

[48] Wang J (2015). Notch Signaling Pathway Regulates Progesterone Secretion in Murine Luteal Cells. Reprod Sci.

[49] George RM, Hahn KL, Rawls A, Viger RS, Wilson-Rawls J (2015). Notch signaling represses GATA4-induced expression of genes involved in steroid biosynthesis. Reproduction.

[50] Frotscher M (2010). Role for Reelin in stabilizing cortical architecture. Trends Neurosci.

[51] Chen J (2001). Cofilin/ADF is required for cell motility during Drosophila ovary development and oogenesis. Nat Cell Biol.

[52] Takahashi T, Koshimizu U, Abe H, Obinata T, Nakamura T (2001). Functional involvement of Xenopus LIM kinases in progression of oocyte maturation. Dev Biol.

[53] Ono K, Ono S (2014). Two actin-interacting protein 1 isoforms function redundantly in the somatic gonad and are essential for reproduction in Caenorhabditis elegans. Cytoskeleton (Hoboken).

[54] Karlsson AB (2010). Luteinizing hormone receptor-stimulated progesterone production by preovulatory granulosa cells requires protein kinase A-dependent activation/dephosphorylation of the actin dynamizing protein cofilin. Mol Endocrinol.

[55] Sisco B, Hagemann LJ, Shelling AN, Pfeffer PL (2003). Isolation of genes differentially expressed in dominant and subordinate bovine follicles. Endocrinology.

[56] Zhang T (2014). Obesity occurring in apolipoprotein E-knockout mice has mild effects on fertility. Reproduction.

[57] Sibbe M, Forster E, Basak O, Taylor V, Frotscher M (2009). Reelin and Notch1 cooperate in the development of the dentate gyrus. J Neurosci.

[58] Kramár EA (2009). Cytoskeletal changes underlie estrogen’s acute effects on synaptic transmission and plasticity. J Neurosci.

[59] Vierk R (2012). Aromatase inhibition abolishes LTP generation in female but not in male mice. J Neurosci.

[60] Okubo T, Suzuki T, Yokoyama Y, Kano K, Kano I (2003). Estimation of estrogenic and anti-estrogenic activities of some phthalate diesters and monoesters by MCF-7 cell proliferation assay *in vitro*. Biol Pharm Bull.

[61] Mendez P, Garcia-Segura LM (2006). Phosphatidylinositol 3-kinase and glycogen synthase kinase 3 regulate estrogen receptor-mediated transcription in neuronal cells. Endocrinology.

[62] McLean, A. C., Valenzuela, N., Fai, S. & Bennett, S. A. Performing vaginal lavage, crystal violet staining, and vaginal cytological evaluation for mouse estrous cycle staging identification. *Journal of visualized experiments: JoVE*, e4389, 10.3791/4389 (2012). 10.3791/4389 PMC3490233 23007862

[63] Hoglund A (1972). A staining method for vaginal smears. Int J Fertil.

[64] Nishi Y (2001). Establishment and characterization of a steroidogenic human granulosa-like tumor cell line, KGN, that expresses functional follicle-stimulating hormone receptor. Endocrinology.

[65] D’Arcangelo G (1997). Reelin is a secreted glycoprotein recognized by the CR-50 monoclonal antibody. J Neurosci.

[66] Forster E (2002). Reelin, Disabled 1, and beta 1 integrins are required for the formation of the radial glial scaffold in the hippocampus. Proc Natl Acad Sci USA.

[67] Garcia-Segura, L. M. *et al*. Aromatase expression by astrocytes after brain injury: implications for local estrogen formation in brain repair. *Neuroscience* **89**, 567–578, S0306-4522(98)00340-6 (1999). 10.1016/s0306-4522(98)00340-6 10077336

[68] Yague JG (2006). Aromatase expression in the human temporal cortex. Neuroscience.

[69] Yague JG (2008). Aromatase distribution in the monkey temporal neocortex and hippocampus. Brain Res.

[70] Livak KJ, Schmittgen TD (2001). Analysis of relative gene expression data using real-time quantitative PCR and the 2(−Delta Delta C(T)) Method. Methods.

[71] Haas CA, Hollerbach E, Deller T, Naumann T, Frotscher M (2000). Up-regulation of growth-associated protein 43 mRNA in rat medial septum neurons axotomized by fimbria-fornix transection. Eur J Neurosci.

