# Supplementary material for: Reelin and aromatase cooperate in ovarian follicle development
Source: Sci Rep. 2018 Jun 7;8:8722. doi: 10.1038/s41598-018-26928-x (PMC5992190; doi:10.1038/s41598-018-26928-x)
Supplement: Supplementary file 1 — Supplementary Dataset 1 [file 41598_2018_26928_MOESM1_ESM.docx]

**Supplemental data:**

**Reelin and aromatase cooperate in ovarian follicle development**

Maurice Meseke, Felicitas Pröls^+^, Camilla Schmahl, Katja Seebo, Claas Kruse, Nicola Brandt, Lars Fester, Lepu Zhou, Gabriele M. Rune^,*^

Institute of Neuroanatomy, University Medical Center Hamburg-Eppendorf, Martinistr. 52, 20246 Hamburg, Germany; **^+^** present address: Institute of Anatomy II, University Medical Department of Cologne, Joseph-Stelzmann Str. 9, 50931 Köln, Germany

Supplemental data to figure 2d:


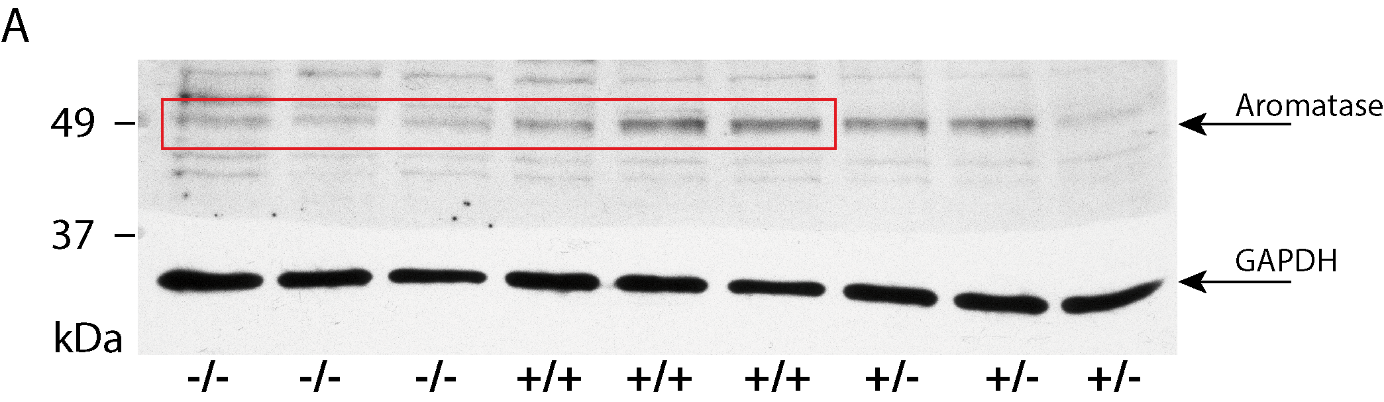


Figure legend:

Figure (A) depicts the original, uncropped western blot presented in the manuscript in figure 2d. Red rectangle highlights the three wild-type (**+/+**) and three homozygous reeler (**-/-**) mice aromatase positive bands of approximately 55 kDa that are presented in the manuscript. Upper black arrow marks the CYP19A1 (aromatase) positive band, lower black arrow the GAPDH positive band used for standardization. Even visual inspection reveals a decreased aromatase positive signal for all three reeler mutants. **+/-** marks the reelin heterozygous genotype, which has not been analyzed.

Supplemental data to figure 4f:


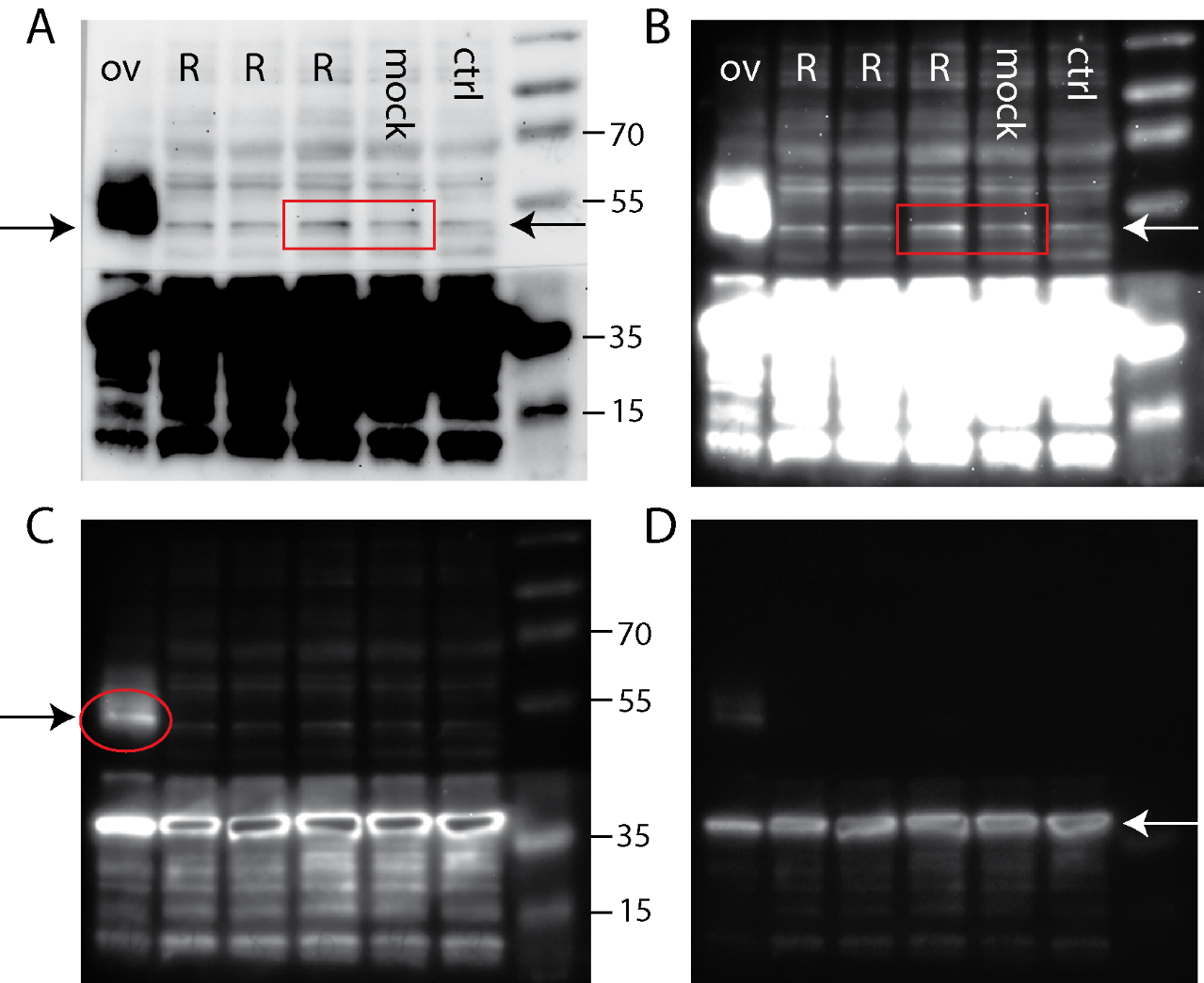


Figure legend:

The original, uncropped version of the presented western blotting bands from figure 4f are shown here.

(A) The overlay and the original picture (B) of a long exposure time demonstrating the increase of CYP19A1 (aromatase) positive bands (black arrow in (A) and white arrow in (B)) in KGN cells after treatment with reelin (R) compared to mock control (mock) or untreated control (ctrl). Red rectangle highlights the aromatase positive bands chosen for presentation in the manuscript. These aromatase positive bands were mirrored in figure 4f of the manuscript to keep the order to present first the control and after that the reelin probe. An independent ovarian probe (ov) from another animal was used as positive control to confirm the correct height of the aromatase band. (C) The ovarian aromatase band presented at a lower exposure time (red circle, black arrow) demonstrating the correct height. (D) GAPDH positive bands for standardization presented at the shortest exposure time (white arrow).
